# Structural characterization and anti-oxidation activity evaluation of pectin from *Lonicera japonica* Thunb.

**DOI:** 10.3389/fnut.2022.998462

**Published:** 2022-09-20

**Authors:** Xiaodan Qi, Yang Yu, Xinyi Wang, Jialei Xu, Xiang Wang, Zhangkai Feng, Yifa Zhou, Hongxing Xiao, Lin Sun

**Affiliations:** ^1^School of Life Sciences, Northeast Normal University, Changchun, China; ^2^Department of Clinical Biochemistry, Qiqihar Medical University, Qiqihar, China

**Keywords:** *Lonicera japonica* Thunb., pectin, endo-polygalacturonase, structural characterization, antioxidant activity

## Abstract

Pectins are nutrient components of plants and are widely used in the food industry. In this study, one major pectin fraction (WLJP-A0.2b) with Mw of 40.6 kDa was purified from *Lonicera japonica* Thunb. The structural feature and antioxidant activity of it was investigated. Monosaccharide composition, Fourier transform infrared (FT-IR) spectra, enzymatic hydrolysis, and nuclear magnetic resonance (NMR) spectra analysis indicated that WLJP-A0.2b consisted of rhamnogalacturonan I (RG-I), rhamnogalacturonan II (RG-II), and homogalacturonan (HG) domains, with mass ratio of 0.4:1.0:2.1. The RG-I domain contained highly branched α-L-1,5-arabinan, β-D-1,4-galactan and type II arabinogalactan (AG-II) side chains. The HG domain was released in the form of un-esterified and partly methyl-esterified and/or acetyl-esterified oligogalacturonides with degree of polymerization 1–8 after degradation by endo-polygalacturonase. Radical scavenging assays indicated that WLJP-A0.2b exhibited antioxidant activity through the synergistic effects of different pectin domains. Oligogalacturonides, especially de-esterified oligogalacturonides, showed better antioxidant activities than RG-II and RG-I domains. Moreover, de-esterified oligogalacturonides remarkably reduced H_2_O_2_-induced reactive oxygen species production in HEK-293T cells. These results provide useful information for screening of natural antioxidants from *Lonicera japonica* Thunb. and application of pectin in functional food field.

## Introduction

*Lonicera japonica* Thunb. (family: Caprifoliaceae) is native to East Asian countries and recognized as edible and medicinal food in daily life (1, 2). The edible flowers and buds are usually used in healthy beverage to improve physical performance and prevent disease in the form of tea, liquor, beverages, acidophilous milk, and oral liquids (3, 4). In the past decades, most of the studies focused on chlorogenic acid, essential oils, and iridoids in *Lonicera japonica* Thunb. In recent years, polysaccharides from *Lonicera japonica* Thunb. have attracted considerable attention due to their diverse biological activities such as anti-tumor, immunomodulation, anti-inflammatory, anti-oxidant, and other biological activities (5–10). Pectin is a family of galacturonic acid-rich polysaccharides found in the primary cell wall of plants (11). It is basically composed of linear homogalacturonan (HG) domain having no side chains, and rhamnogalacturonan I and II (RG-I and RG-II) domains with diverse side chains (12). Some pectin fractions have been purified from *Lonicera japonica* Thunb. Lin et al. isolated a homogenous pectin fraction termed LJ-02-1 from *Lonicera japonica* Thunb. by DEAE-cellulose and Sephacryl S-200HR column. Further analysis revealed that LJ-02-1 had an average molecular weight of 54 kDa and was mainly composed of rhamnose, galacturonic acid, galactose, and arabinose in the molar ratio of 10.77:7.88:15.45:65.89 (5). Liu et al. isolated a pectic polysaccharide (LFA03-a) from *Lonicera japonica* Thunb. Structural analysis revealed that it possessed a RG-I backbone and substituted at O-4 of L-rhamnose with β-D-1,4-Galp, β-D-1,3-Galp, β-D-1,3,6-Galp, and α-L-1,5-Araf side chains (6). In addition, Zhang et al. purified one neutral fraction (LJP-N) and four acidic fractions (LJP-A-1 ∼ LJP-A-4) from *Lonicera japonica* Thunb. Among these fractions, LJP-A-3 was defined as HG-type pectin with a trace of RG-I domain which exhibited antioxidant activity (9). Although the chemical structures of a few pectin fractions from *Lonicera japonica* Thunb. have been analyzed, their detailed structures remain unknown, and their structure-activity relationships need to be further investigated.

Oxidative stress or an elevated level of reactive oxygen species (ROS) can induce oxidative damage, and consequently cause the occurrence and development of diseases like diabetes mellitus (13), cancer (14), cardiovascular (15), Alzheimer’s disease (16), inflammatory diseases (17), aging (18), etc. Therefore, finding suitable antioxidants to neutralize free radicals is of great importance to protect cells from excessive oxidative stress. In recent years, pectic polysaccharides have been widely reported to exhibit antioxidant activities. Pectin can be viewed as a carrier of hydrogen which could bind with free radicals to terminate free radical chain reaction. Pectin derived from edible and medicinal plants is safe and healthy, therefore, it is a new type of natural antioxidant. The antioxidant activities of pectins from different sources depend on their structural characteristics, however, the structure-antioxidant activity relationship of pectin remain unclear.

In the present study, pectin polysaccharides were extracted from *Lonicera japonica* Thunb., systematically fractionated and structurally characterized. Moreover, pectin domains were prepared by enzymatic hydrolysis and the structure and antioxidant activities of different domains were intensively studied. These findings provide new information on structure-antioxidant activity relationships of pectins from *Lonicera japonica* Thunb., and facilitate their utilization in the field of functional food.

## Materials and methods

### Materials and reagents

*Lonicera japonica* Thunb. was purchased from Henan Province, China. DEAE-Cellulose was acquired from Shanghai Chemical Reagent Research Institute (Shanghai, China). Sepharose CL-6B and Sephadex G-75 were obtained from GE Healthcare (Pittsburgh, PA, United States). Endo-polygalacturonase (Endo-PG) was purchased from Megazyme (Bray, Ireland). All other chemicals were of analytical or HPLC grade made in China.

### General methods

Total carbohydrate and uronic acid contents were measured by phenol-sulfuric acid and meta-hydroxydiphenyl methods, using the mixture of major monosaccharides and galacturonic acid (GalA) as standards independently (19, 20). Starch-like polysaccharide was determined by using the I_2_–KI method. Kdo and Dha are the component of RG-II, which was qualitatively detected by using the modified thiobarbituric acid (TBA) method (21). Protein content was determined by using the Bradford assay with bovine serum albumin (BSA) as standard (22). Homogeneity and molecular weight (Mw) were determined by using high performance gel-permeation chromatography (HPGPC) with a TSK-gel G-3000 PWXL column (7.8 × 300 mm, TOSOH, Japan) coupled to a Shimadzu high performance liquid chromatography (HPLC) system (Tokyo, Japan) (23). Monosaccharide composition was analyzed by using HPLC and PMP pre-column derivatization method as previously described (24). Fourier transform infrared (FT-IR) spectra was recorded over a range of 4000 and 500 cm^–1^ using a PerkinElmer Spectrum Two FT-IR spectrometer (Perkin Elmer, United States).

### Extraction of polysaccharides from *Lonicerae japonicae* Thunb.

*Lonicerae Japonicae* Thunb. (1 kg) was washed to remove the dust and immersed in 16 L distilled water overnight, and then extracted at 100°C for 3 h. After filtration, the residues were extracted twice again under the same conditions. The filtrates were combined, concentrated, and then centrifuged (4500 rpm × 15 min). The supernatant was precipitated overnight by the addition of 95% ethanol (three volumes). The precipitates were obtained by centrifugation (4500 rpm × 15 min) and washed with 95% ethanol and anhydrous ethanol, and then dried at room temperature. The water-soluble *Lonicerae Japonicae* Thunb. polysaccharides was obtained and named WLJP.

### Purification of pectin from WLJP

WLJP (60 g) was dissolved in distilled water (50 mg/ml) and applied to a DEAE-Cellulose column. It was eluted with distilled water to give the un-bound fraction WLJP-N and then with 0.5 M NaCl to give the bound fraction WLJP-A. WLJP-A (20 g) was then applied to DEAE-Cellulose column, and eluted stepwise with distilled water, 0.2, 0.3, and 0.5 M NaCl to produce four fractions WLJP-AH, WLJP-A0.2, WLJP-A0.3, and WLJP-A0.5. The major fraction WLJP-A0.2 was further purified by using a Sepharose CL-6B column (3.0 × 90 cm), and two sub-fractions WLJP-A0.2a and WLJP-A0.2b were obtained, with the latter as the major pectic fraction.

### Enzymatic hydrolysis and de-esterification

WLJP-A0.2b was dissolved in HAc-NaAc buffer solution (50 mM, pH 4.5) and incubated with Endo-PG at 40°C for 24 h. The enzyme was inactivated by heating at 100°C for 10 min and removed by centrifugation (12000 rpm × 5 min). The hydrolysate was fractionated on a Sephadex G-75 (3.0 × 90 cm) column and eluted with 0.15 M NaCl at 0.4 ml/min. The eluates (8 ml per tube) were collected and assayed for distribution of total carbohydrate and uronic acid contents. The appropriate fractions were combined, desalted (1 kDa cutoff dialysis tubes or a Sephadex G-10 column) and freeze-dried to give three sub-fractions WLJP-A0.2b-E1∼E3.

The de-esterification was performed according to the previous method (25). WLJP-A0.2b-E3 was saponified by 0.1 M NaOH and held at 4°C for 4 h with mild stirring. The reaction solutions were neutralized with 10% glacial acetic acid, and de-esterified pectin fractions were obtained and called D-WLJP-A0.2b-E3.

### Nuclear magnetic resonance spectroscopy

^13^C, Heteronuclear Single Quantum Coherence (HSQC), and Heteronuclear Multiple Bond Correlation (HMBC) NMR spectra were recorded at 25°C on a Bruker Avance 600 MHz spectrometer (Bruker, Karlsruhe, Germany) with a Bruker 5 mm broadband probe. Samples (20 mg) were dissolved in D_2_O (99.8%, 0.5 ml) and centrifuged to remove undissolved components. Chemical shifts were given in ppm, with acetone as an internal chemical shift reference. Data were analyzed using standard Bruker software.

### ESI-MS analysis

Oligosaccharide fraction (1 mg/ml in 50% (v/v) acetonitrile) was analyzed by electrospray ionization-mass spectrometry (ESI-MS) using an Amazon ETD Ion Trap Mass Spectrometer (Bruker Inc., Germany). The sample was delivered to electrospray source using a syringe pump at a flow rate of 5 μL/min. ESI-MS detection was performed in negative mode with capillary voltage 3500 V, capillary temperature 200°C and dry gas 2 ml/min. The mass scan range was *m*/*z* 100 to 2000. Data were processed using Trap-control software.

### *In vitro* antioxidant activity

#### ABTS radical scavenging activity

The scavenging capacity for 2,2′-Azinobis-(3-ethylbenzthiazoline-6-sulphonate) (ABTS) free radical was measured following previous research (26), with some modifications. Solutions of ABTS (7 mM) and potassium persulfate (2.45 mM) were mixed and incubated in the dark at room temperature for 12–16 h to prepare ABTS radical. In the moment of use, the ABTS radical solution was diluted with phosphate-buffered saline (0.1 M PBS, pH 7.4) to obtain an absorbance of 0.70 (±0.05) at 734 nm. 50 μL of pectin samples solution at five concentrations (0.5, 1.0, 2.0, 5.0, and 10.0 mg/ml) and 500 μL of the diluted ABTS radical solution were mixed evenly and incubated at 37°C for 30 min. The absorbance was measured at 734 nm. Distilled water was used as blank control, and ascorbic acid (V_*C*_) was used as positive control. Each group was tested for three times in parallel. ABTS radical scavenging activity was calculated according to the following equation:


$$\text{Scavengingactivity}\left(\%\right)\text{ = }\left[1-\left({\text{A}}_{\text{x}}-{\text{A}}_{\text{x0}}\right){\text{/A}}_{\text{0}}\right]\times 100\%$$

A_0_: The absorbance value of blank control;

A_*x*_: The absorbance value of sample solution;

A_*x*0_: The absorbance value of the sample background (PBS instead of ABTS radical solution).

#### Hydroxyl radical scavenging activity

The hydroxyl radical scavenging activity was determined as previously reported (27), with some modifications. 50 μL of pectin samples solution at five concentrations (0.5, 1.0, 2.0, 5.0, and 10.0 mg/ml), 100 μL of 9 mM FeSO_4_, 100 μL of 9 mM anhydrous ethanol-salicylic acid and 100 μL of 8.8 mM H_2_O_2_ were mixed evenly and incubated at 25°C for 30 min. Then the mixture was centrifuged (12000 rpm × 5 min), and the absorbance of supernatant was measured at 510 nm. Distilled water was used as blank control, and V_*C*_ was used as positive control. Each group was tested for three times in parallel. The hydroxyl radical scavenging activity was calculated according to the formula:


$$\text{Scavengingactivity}\left(\%\right)\text{ = }\left[1-\left({\text{A}}_{\text{x}}-{\text{A}}_{\text{x0}}\right){\text{/A}}_{\text{0}}\right]\times 100\%$$

A_0_: The absorbance value of blank control;

A_*x*_: The absorbance value of sample solution;

A_*x*0_: The absorbance value of the sample background (distilled water instead of H_2_O_2_ solution).

#### DPPH radical scavenging activity

The 1,1-Dihpenyl-2-picrylhydrazyl (DPPH) radical scavenging ability was assessed according to the reported method (28), with some modifications. 50 μL of pectin samples solution at five concentrations (0.5, 1.0, 2.0, 5.0, and 10.0 mg/ml) and 200 μL of 0.004% anhydrous methanol-DPPH solution were mixed evenly and incubated at 25°C for 30 min in the dark. Then the mixture was centrifuged (12000 rpm × 5 min), the absorbance of supernatant was measured at 517 nm. Distilled water was used as blank control, and V_*C*_ was used as positive control. Each group was tested for three times in parallel. The DPPH radical scavenging activity was calculated according to the formula:


$$\text{Scavengingactivity}\left(\%\right)\text{ = }\left[1-\left({\text{A}}_{\text{x}}-{\text{A}}_{\text{x0}}\right){\text{/A}}_{\text{0}}\right]\times 100\%$$

A_0_: The absorbance value of blank control;

A_*x*_: The absorbance value of sample solution;

A_*x*0_: The absorbance value of the sample background (anhydrous methanol instead of DPPH solution).

### Antioxidant activity against H_2_O_2_ induced oxidative stress in HEK-293T cells

#### Cell culture and treatment

HEK-293T cells were provided by Cell Resource Center, Institute of Basic Medical Sciences, Chinese Academy of Medical Sciences (Beijing, China). The cells were cultured in DMEM medium supplemented with 10% fetal bovine serum (Clark), 100 U/ml penicillin and 100 μg/ml streptomycin at 37°C in 5% CO_2_. The cells were plated in 6-well plates at a density of 1 × 10^5^ cells per well before experimentation. Adherent cells were pretreated with indicated concentrations (10, 50, and 100 μg/ml) of de-esterified pectin samples (D-WLJP-A0.2b-E3) for 24 h, respectively, and then treated with H_2_O_2_ (200 μm) for 6 h.

#### Reactive oxygen species measurement

The reagent 2, 7-dichlorodihydrofluorescein diacetate (DCFH-DA) was used to determine the intracellular levels of ROS. After pretreatment, the cells were harvested and washed with phosphate-buffered saline (PBS). Then the cells were incubated with 10 mM DCFH-DA (Sigma-Aldrich, Saint Louis, MO, United States) for 30 min at 37°C in dark. After DCFH-DA was removed, the cells were washed twice with PBS and re-suspended for detection with flow cytometer (BD Biosciences, United States) (29).

### Statistical analysis

All experiments were performed in triplicate, and the data were shown as the mean ± standard deviation (SD). Student’s *t*-test was used for single comparison. A value of *p* < 0.05 was considered statistically significant. IC_50_ values were calculated using SPSS 19.0 software.

## Results and discussion

### Preparation of pectin from *Lonicerae japonicae* Thunb.

Water-soluble *Lonicerae Japonicae* Thunb. polysaccharide (WLJP) was obtained following hot water extraction and ethanol precipitation, with the yield of 7.6%. Monosaccharide composition analysis (Table 1) showed that WLJP mainly consisted of GalA (30.9%), Ara (30.9%), Gal (15.9%), and Glc (14.2%), with minor Rha (4.5%), Man (1.9%), Xyl (1.2%), and GlcA (0.5%). The I2-KI assay indicated that it contained small amounts of starch-like polysaccharide.

**TABLE 1:** Yields, Mws, and monosaccharide compositions of polysaccharide fractions from *Lonicerae japonicae* Thunb.

Fraction	Yield (w%)	Mw (kDa)	TBA	Monosaccharide composition (mol%)							
				GalA	Rha	Gal	Ara	Glc	GlcA	Xyl	Man
WLJP				30.9	4.5	15.9	30.9	14.2	0.5	1.2	1.9
WLJP-N	34.2^a^			2.9	4.9	25.3	33.7	22.9	2.8	2.0	5.5
WLJP-A	31.6^a^			50.4	7.0	10.8	26.2	4.0	−	1.6	−
WLJP-A0.2b	11.1^a^	40.6		72.2	2.8	5.8	15.9	2.0	0.5	0.6	0.2
WLJP-A0.2b-E1	7.6^b^	90.0	-	10.3	7.5	27.8	50.7	3.7	−	−	−
WLJP-A0.2b-E2	20.8^b^	14.1	+	70.8	9.4	4.6	10.7	2.2	1.4	−	0.9
WLJP-A0.2b-E3	43.0^b^	4.7	-	90.5	6.6	1.1	1.0	0.8	−	−	−

^a^Yield in relation to WLJP.

^b^Yield in relation to WLJP-A0.2b.

**FIGURE 1:**
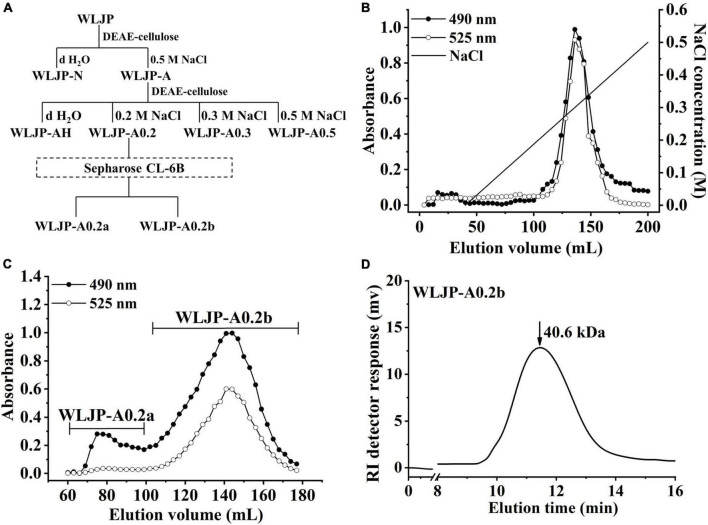
Preparation of pectin from *Lonicerae japonicae* Thunb. **(A)** Fractionation scheme of WLJP by anion-exchange and size-exclusion chromatographies. **(B)** Linear gradient elution profile of WLJP-A on DEAE-cellulose column. **(C)** Elution profile of WLJP-A0.2 on Sepharose CL-6B column. (-🌑- total carbohydrate; -○- uronic acid). **(D)** Molecular weight distribution of WLJP-A0.2b by high performance gel-permeation chromatography (HPGPC) analysis.

WLJP was fractionated by using anion-exchange and size-exclusion chromatographies (Figure 1A). First, WLJP was separated by anion exchange chromatography into neutral polysaccharide fraction WLJP-N and acidic polysaccharide fraction WLJP-A. WLJP-N contained little GalA, and thus was not further discussed in this paper. WLJP-A is mainly composed of GalA (50.4%), typical for pectin. The charge distribution of WLJP-A was not homogeneous (Figure 1B). Therefore, WLJP-A was further separated by using anion exchange chromatography, and four fractions WLJP-AH (yield 1.2%), WLJP-A0.2 (yield 71.8%), WLJP-A0.3 (yield 9.7%), and WLJP-A0.5 (yield 2.5%) were obtained. The main fraction WLJP-A0.2 was further purified by size exclusion chromatography (Figure 1C), and the major pectic fraction WLJP-A0.2b was obtained. WLJP-A0.2b showed single symmetrical peak on HPGPC (Figure 1D) with molecular weight of 40.6 kDa. It was mainly composed of GalA (72.2%), with minor Ara (15.9%), Gal (5.8%), and Rha (2.8%) (Table 1). The structure of WLJP-A0.2b will be analyzed in detail in this study.

### Structural analysis of WLJP-A0.2b

#### Fourier transform infrared analysis of WLJP-A0.2b

**FIGURE 2:**
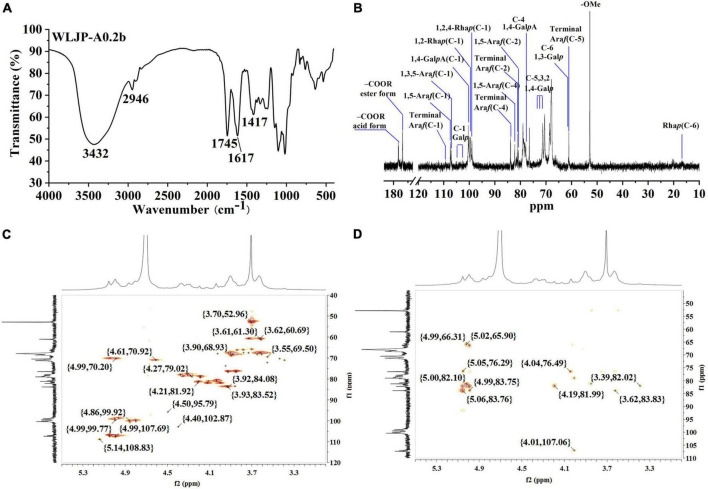
Structural analysis of WLJP-A0.2b. **(A)** Fourier transform infrared (FT-IR) spectrum. **(B)** ^13^C nuclear magnetic resonance (NMR) spectrum. **(C)** HSQC spectrum. **(D)** HMBC spectrum.

The FT-IR spectra of WLJP-A0.2b was presented in Figure 2A. The typical broad peak near 3432 cm^–1^ was assigned to the stretching vibration of O-H, and the adsorption around 2946 cm^–1^ was attributed to C–H stretching of CH_2_ groups (30). The bands around 1745, 1617, and 1417 cm^–1^ confirmed the presence of uronic acid. The peaks at around 1745 and 1617 cm^–1^ represent methyl-esterified carbonyl stretching (COOR) and ionic carboxyl band (COO-), respectively (31). The degree of methyl-esterification (DM) was calculated by the area of the band at 1745 cm^–1^ to the sum of the areas of the bands at 1745 and 1617 cm^–1^ (32). The result showed that the DM of WLJP-A0.2b was 32.9%, which was low esterification pectin. The stretching bands between 1010 and 1150 cm^–1^ (e.g., 1018, 1103, and 1145 cm^–1^) corresponded to the absorption of C-O and C-C glycosidic bonds and the pyranose rings (33). In addition, the peaks around 858 and 896 cm^–1^ suggested the presence of α-linked and β-linked glycosyl residues, respectively (28).

#### Nuclear magnetic resonance analysis of WLJP-A0.2b

Structural feature of WLJP-A0.2b was characterized by ^13^C NMR spectrum (Figure 2B). The chemical shifts were assigned predominantly according to ^1^H and ^13^C correlations in the HSQC (Figure 2C) and HMBC spectra (Figure 2D).

The typical resonance signals at 100.05/4.80, 68.59/3.60, 70.46/3.90, 77.87/4.31, 70.63/4.61, and 174.91 ppm were assigned to C-1/H-1 to C-5/H-5 and C-6 of unesterified 1,4-α-D-Gal*p*A, respectively, suggesting the presence of HG-type pectin. The signal at 170.68 and 52.77 ppm were attributed to C-6 of methyl-esterified 1,4-α-D-Gal*p*A and methyl groups, respectively. The weak signal at 20.03/2.07 ppm was attributed to acetyl group attached to α-D-Gal*p*A. The resonance signals at 16.81/1.21 and 16.22/1.16 ppm were assigned to C-6/H-6 of 1,2,4-α-L-Rha*p* and 1,2-α-L-Rha*p*, respectively, indicating the presence of RG-I type pectin. The anomeric signals at 108.98/5.14, 107.38/4.99, and 107.06/5.04 ppm corresponded to C-1/H-1 of terminal α-L-Ara*f*, 1,5-α-L-Ara*f*, and 1,3,5-α-L-Ara*f*, respectively. Signals at 104.05/4.40, 103.35/4.37, 103.35/4.37, 103.18/4.35, and 102.31/4.28 ppm were attributed to C-1/H-1 of 1,4-β-D-Gal*p*, 1,3,6-β-D-Gal*p*, terminal β-D-Gal*p*, 1,6-β-D-Gal*p*, and 1,3-β-D-Gal*p*, respectively. The complex and over-lapped signals at 60 ppm-85 ppm were assigned to C-2 to C-5 of different linkages of Rha, Ara and GalA, and C-2 to C-6 of β-D-Gal*p*. These chemical shift assignments were listed in Supplementary Table 1. Additionally, some characteristic signals representing for RG-II type pectin were also found. Signals at 107.36/4.98, 103.38/4.40, and 96.53/4.50 ppm were attributed to C-1/H-1 of α-Ara*f*, α-Ara*p*, and 2-O-Me-α-Xyl*p*, respectively. Signals at 95.11 and 91.87 ppm were assigned to C-2 of α-Kdo*p* and α-Ace*f*A (34, 35). NMR analysis showed that WLJP-A0.2b was composed of HG, RG-I, and RG-II domains. Further analysis of HSQC and HMBC spectra revealed that WLJP-A0.2b contained terminal α-L-Ara*f*, α-L-1,5-Ara*f*, α-L1,3,5-Ara*f*, and β-D-1,3,6-Gal*p* glycosyl linkages, which might form α-L-1,5-arabinan and AG-II side chains in RG-I.

#### Enzymatic analysis of WLJP-A0.2b

**FIGURE 3:**
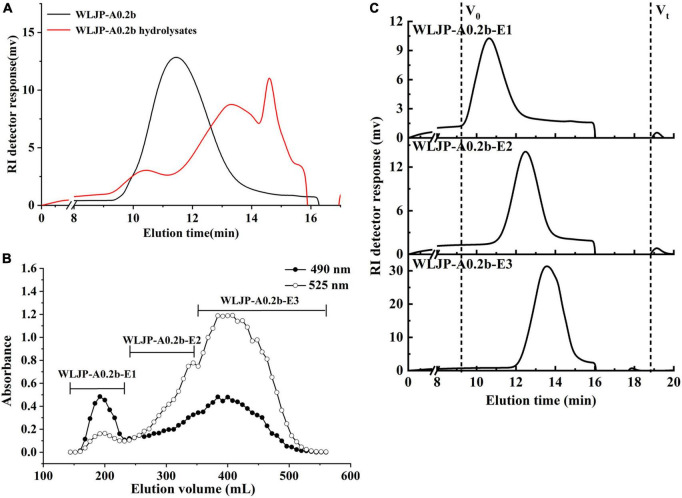
**(A)** The molecular weight distributions of WLJP-A0.2b before and after endo-polygalacturonanase (Endo-PG) hydrolysis. **(B)** The elution profile of enzymatic hydrolysis product of WLJP-A0.2b on Sephadex G-75 (-🌑- total carbohydrate; -○- uronic acid). **(C)** High performance gel-permeation chromatography (HPGPC) elution profiles of three sub-fractions of WLJP-A0.2b.

Enzymatic degradation is an efficient tool for elucidating the structure of pectin. To further analyze the structure of WLJP-A0.2b, it was degraded by Endo-PG which could hydrolyze at least four continuous un-esterified α-(1-4)-GalA in HG to release different pectin domains (36, 37). After degradation of WLJP-A0.2b by Endo-PG, the product was detected by HPGPC (Figure 3A). As can be seen, the molecular weight distribution of WLJP-A0.2b changed significantly after enzymatic hydrolysis. After separation by size-exclusion chromatography, three sub-fractions (WLJP-A0.2b-E1, WLJP-A0.2b-E2, and WLJP-A0.2b-E3) were obtained, with yield of 7.6, 20.8, and 43.0%, respectively (Figure 3B).

WLJP-A0.2b-E1 contained Gal, Ara, Rha, and GalA as major monosaccharides (Table 1), and the molar ratio of Rha/GalA was 0.7, typical for RG-I type pectin (38). WLJP-A0.2b-E1 showed single symmetrical peaks on HPGPC with a TSK-gel G-3000 PW_*XL*_ column, and the molecular weight of it was estimated to be 90.0 kDa (Figure 3C). WLJP-A0.2b-E2 was mainly composed of GalA (70.8%), and the molecular weight of it was 14.1 kDa. TBA assay showed positive result, indicating WLJP-A0.2b-E2 was RG-II type pectin. WLJP-A0.2b-E3 contained more than 90% GalA, with molecular weight of 4.7 kDa, indicating that it was oligogalacturonide produced by Endo-PG hydrolyzing of HG domains.

Based on these results, we concluded that WLJP-A0.2b was composed of RG-I, RG-II, and HG domains with mass ratio of 0.4:1.0:2.1, and HG was the dominant domain in WLJP-A0.2b. Different domains were covalently linked and released after Endo-PG degradation (27). As RG-II is a type of structurally conserved pectin, it was not further analyzed in this study. We continued to investigate the structures of RG-I and oligogalacturonides.

### Structural analysis of pectin domains from WLJP-A0.2b

#### Nuclear magnetic resonance analysis of rhamnogalacturonan I domain

**FIGURE 4:**
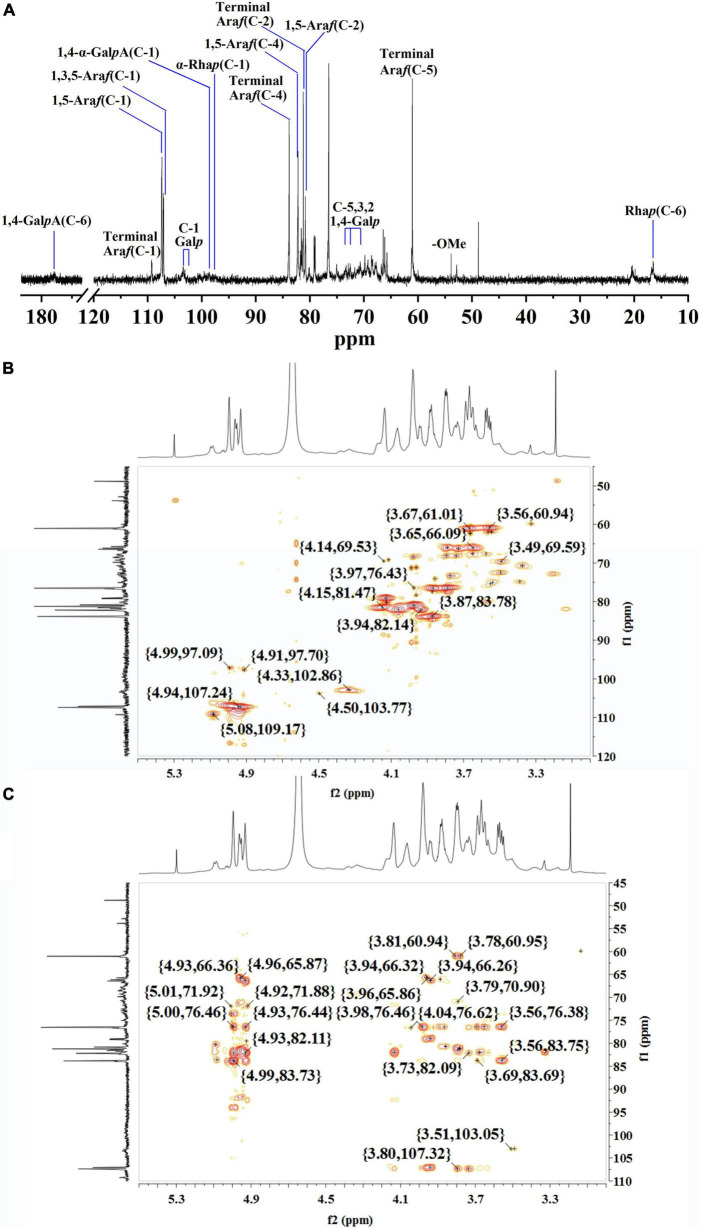
Nuclear magnetic resonance (NMR) spectra of WLJP-A0.2b-E1. **(A)** ^13^C NMR spectrum. **(B)** HSQC spectrum. **(C)** HMBC spectrum.

The structure of WLJP-A0.2b-E1 was analyzed by ^13^C NMR spectrum (Figure 4A). ^1^H and ^13^C signals were assigned by using HSQC (Figure 4B) and HMBC spectra (Figure 4C). The assignments of the major chemical shifts were listed in Supplementary Table 2. The signals at 173.64 and 98.60 ppm were attributed to C-6 and C-1 of 1,4-α-D-Gal*p*A, respectively. The resonance signals at 16.46/1.08 and 16.78/1.15 ppm were assigned to C-6/H-6 of 1,2-α-L-Rha*p* and 1,2,4-α-L-Rha*p*, with their corresponding anomeric resonances at 97.47/5.00 ppm. These signals indicated WLJP-A0.2b-E1 contained RG-I backbone. The anomeric signals at 109.26/5.08, 107.38/4.93, and 107.06/4.94 ppm were attributed to C-1/H-1 of terminal α-L-Ara*f*, 1,5-α-L-Ara*f*, and 1,3,5-α-L-Ara*f*, respectively. The anomeric signal at 102.64/4.33 ppm and the corresponding C-6/H-6 signal at 59.75/3.56 ppm were attributed to terminal β-D-Gal*p*. The C-1/H-1 signals at 103.38/4.50, 103.10/4.49, 102.64/4.33, and 102.60/4.32 ppm, and the corresponding C-6/H-6 signals at 59.96/3.55, 69.23/4.11, 69.23/4.11, and 61.18/3.67 ppm were assigned to 1,4-β-D-Gal*p*, 1,3,6-β-D-Gal*p*, 1,6-β-D-Gal*p*, and 1,3-β-D-Gal*p*, respectively. The typical signal at 76.68/3.87 ppm was assigned to C-4/H-4 of 1,4-β-D-Gal*p.* NMR analysis result indicated that WLJP-A0.2b-E1 possessed α-L-1,5-arabinan, β-D-1,4-galactan and AG-II side chains in RG-I domain. As signals corresponding to 1,3,4-β-D-Gal*p* or 1,4,6-β-D-Gal*p* were not clearly observed, AG-I side chains might be not present in WLJP-A0.2b-E1 (39).

#### ESI-MS analysis of oligogalacturonides

**FIGURE 5:**
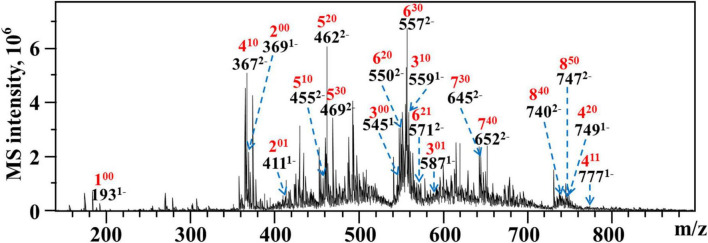
ESI-MS analysis of WLJP-A0.2b-E3. Peak annotation: 4^20^, DP 4, 2 *O*-methyl-ester group, 0 *O*-acetyl group.

As WLJP-A0.2b-E3 was oligogalacturonide, the structure of it was further analyzed by ESI-MS (Figure 5). An overview of *m*/*z* values and speculated structures were shown in Supplementary Table 3.

ESI-MS result indicated that WLJP-A0.2b-E3 was composed of un-esterified and partly methyl-esterified and/or acetylated oligogalacturonides with degree of polymerization (DP) from 1 to 8. The monomer (1^00^), dimer (2^00^), and trimer (3^00^) were un-esterified, while other oligogalacturonides were methyl-esterified and/or acetylated. It was possible that the presence of esterification in HG domain caused the resistance of Endo-PG, which acts on four continuous un-esterified α-(1,4)-GalA linkages (40). In WLJP-A0.2b-E3, GalA dimers, trimers, and tetramers containing one to two methyl-esters and/or one acetyl group were detected (2^01^, 3^01^, 4^10^, 4^11^, and 4^20^). GalA oligomers with DP 5–6 carrying one to three methyl-ester groups and/or one acetyl group (5^10^, 5^20^, 5^30^, 6^20^, 6^21^, and 6^30^) were found. Oligosaccharide fragments with higher DP containing methyl-ester substitutions, e.g., GalA oligomers with DP 7–8 carrying three to five methyl ester groups (7^30^, 7^40^, 8^40^, and 8^50^) were also found.

### *In vitro* antioxidant activity assays

**FIGURE 6:**
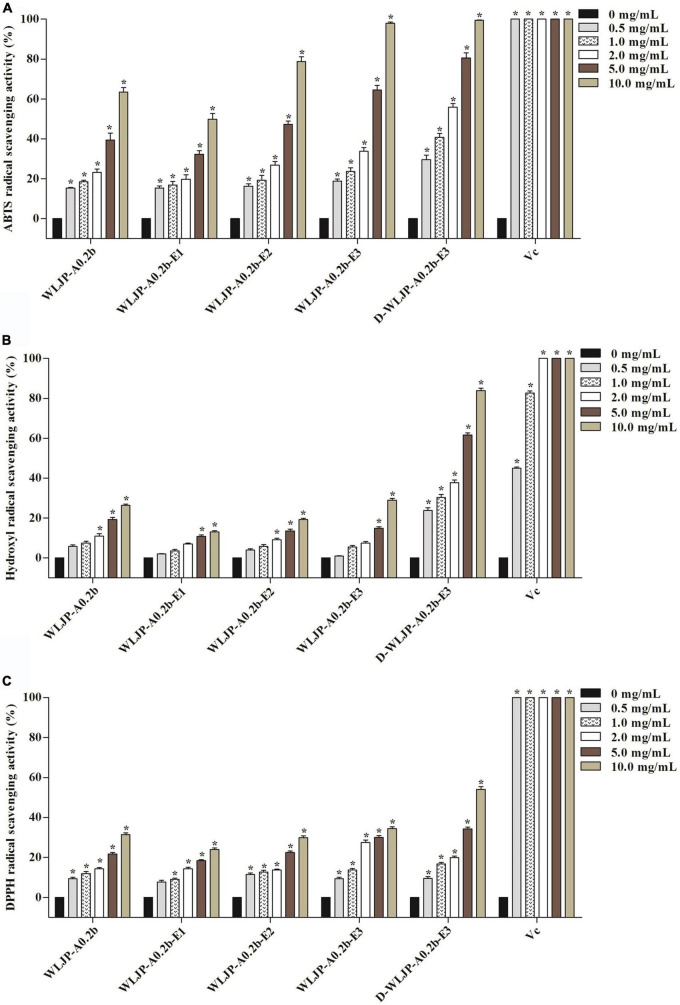
Scavenging abilities of pectin from *Lonicera japonica* Thunb. on **(A)** ABTS radical, **(B)** hydroxyl radical, **(C)** DPPH radical. Vc was used as the positive control. Each value represented the mean ± SD (*n* = 3, *p < 0.05). All experiments were performed in triplicate.

The antioxidant activities of WLJP-A0.2b and its different domains including RG-I (WLJP-A0.2b-E1), RG-II (WLJP-A0.2b-E2), and oligogalacturonides (WLJP-A0.2b-E3) were determined by using ABTS, hydroxyl and DPPH radical scavenging assays. As shown in Figure 6, WLJP-A0.2b exhibited effective antioxidant activities in the three scavenging assays. After degradation by Endo-PG, the pectin domains isolated from WLJP-A0.2b displayed different antioxidant activities. Compared with RG-I and RG-II domains, oligogalacturonides (WLJP-A0.2b-E3) exhibited the highest antioxidant activities. Meanwhile, the antioxidant activity of oligogalacturonides was also higher than that of WLJP-A0.2b. The radical scavenging abilities of oligogalacturonides were dose-dependent with the increase of the concentration from 0.5 to 10 mg/ml, but weaker than that of ascorbic acid (Vc). The IC_50_ values (Table 2) further indicated that the antioxidant activity of oligogalacturonides was better than RG-II and followed by RG-I domains. Similar results have been reported that SPS-3 and SPS-2 from fruit of Actinidia arguta were HG-rich pectin, which displayed higher antioxidant activities than RG-rich pectin (41).

**TABLE 2:** IC_50_ values of different pectins from *Lonicera japonica* Thunb. toward the scavenging of ABTS, hydroxyl, and DPPH radical.

Fractions	IC_50_ values (mg/ml)		
	ABTS	⋅OH^–^	DPPH
WLJP-A0.2b	6.76 ± 0.11	51.69 ± 0.52	53.71 ± 0.58
WLJP-A0.2b-E1	13.63 ± 0.51	182.15 ± 0.87	125.27 ± 0.91
WLJP-A0.2b-E2	4.14 ± 0.13	121.19 ± 0.91	86.81 ± 0.41
WLJP-A0.2b-E3	2.40 ± 0.03	25.14 ± 0.80	27.06 ± 0.53
D-WLJP-A0.2b-E3	1.29 ± 0.08	2.51 ± 0.27	9.41 ± 0.12

The higher antioxidant activities of oligogalacturonides might be related to its lower molecular weight and higher GalA content (42, 43). As shown in Supplementary Figure 1, the IC_50_ of WLJP-A0.2b-E1 to E3 decreased gradually along with the decrease of their molecular weights, while their IC_50_ values decreased with the increase of galacturonic acid content. The possible reason might be that oligogalacturonides with lower molecular weight would have more reductive hydroxyl group terminals (on per unit mass basis) to accept and eliminate the free radicals (44, 45). Moreover, higher amounts of GalA units present more electrophilic groups, which facilitates the liberation of hydrogen from O-H bond, and thus have good hydrogen supply capacity to scavenge the radical (45, 46).

In addition, the degree of methyl-esterification (DM) might be also related to the antioxidant activity of oligogalacturonides (45, 47). ESI-MS result indicated that WLJP-A0.2b-E3 contained mostly methyl-esterified oligogalacturonides. To investigate the relationship between DM and antioxidant activities, WLJP-A0.2b-E3 was saponified by alkali to remove methyl-ester groups which was confirmed by FT-IR spectrum (Supplementary Figure 2). The de-esterified oligogalacturonides named D-WLJP-A0.2b-E3 was then obtained. The molecular weight distributions of D-WLJP-A0.2b-E3 was the same as WLJP-A0.2b-E3 on HPGPC (Supplementary Figure 3). As shown in Figure 6, D-WLJP-A0.2b-E3 showed stronger antioxidant capacity than WLJP-A0.2b-E3. The radical scavenging abilities of it increased dose-dependently with the increase of concentration from 0.5 to 10 mg/ml, but still weaker than Vc. It was supposed that more carboxylic groups and electrophilic groups were exposed after de-esterification which were benefit for antioxidant activity. The result was consistent with previous findings (48).

Based on above analysis, we speculated that the antioxidant activity of WLJP-A0.2b was due to the synergistic effects of different pectin domains. Among different domains, oligogalacturonides derived from HG domain displayed the best antioxidant activities, followed by RG-II domain and then RG-I domain. The structural differences including molecular weight, the content of galacturonic acid and the degree of methyl-esterification in different domains appear to explain their varied activities. The de-esterified oligogalacturonide which contained the highest content of galacturonic acid, the lowest molecular weight and the lowest degree of methyl-esterification might be the reason for its strongest antioxidant activity.

### Oligogalacturonides reduced intracellular reactive oxygen species generation

**FIGURE 7:**
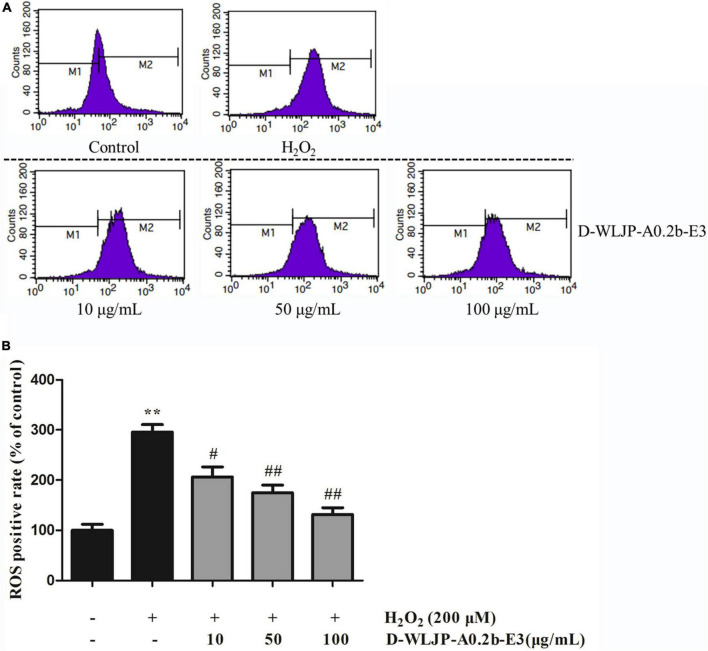
Effects of D-WLJP-A0.2b-E3 treatment at different concentrations on H_2_O_2_-induced intracellular accumulation of reactive oxygen species (ROS) in HEK-293T cells. **(A)** The intracellular levels of ROS analyzed by flow cytometry. **(B)** Histogram analysis of intracellular accumulation of ROS. Data are presented as means ± SD from three independent experiments. **p < 0.01 vs. the control group, ^#^*p* < 0.05 vs. H_2_O_2_ model group, and ^##^*p* < 0.01 vs. H_2_O_2_ model group.

Oxidative stress with the excessive production of ROS is considered as the key factor to damage important cellular components including protein, lipid, and DNA, thus resulting in cytotoxicity (49), even leading to cell apoptosis (50, 51). Hence, scavenging the increased ROS to decrease the oxidative stress level and maintain the balance of cellular redox has become urgent. *In vitro* antioxidant activity assays, we have found that de-esterified oligogalacturonides (D-WLJP-A0.2b-E3) displayed the strongest antioxidant activities. To further assess its antioxidant activities, the level of intracellular ROS production after treatment by the oligogalacturonides was tested by flow cytometry. As shown in Figure 7, ROS levels increased significantly after being injured with H_2_O_2_ (248.49 ± 17.37) in contrast to the control group (117.74 ± 13.63) in HEK-293T cells. However, pretreatment with oligogalacturonides significantly reduced the levels of H_2_O_2_-induced ROS formation with a dose-dependent manner. When the concentration of oligogalacturonides was 100 μg/ml, the ROS level was reduced to 138.60 ± 15.86. These results indicated that oligogalacturonides could protect HEK-293T cells from H_2_O_2_-induced oxidative stress injury by inhibiting ROS generation, and further confirmed the antioxidant activity of oligogalacturonides.

## Conclusion

In the present work, one major pectin fraction WLJP-A0.2b was purified from *Lonicera japonica* Thunb. Structural analysis indicated that it was dominated by HG domains, covalently linked with RG-I and RG-II domains. The mass ratio of RG-I, RG-II, and HG domains in WLJP-A0.2b was 0.4:1.0:2.1. After Endo-PG hydrolysis, HG domain was degraded into oligogalacturonides with DP 1–8 carrying less than 5 methyl-ester groups. RG-I domain from WLJP-A0.2b contained α-L-1,5-arabinan, β-D-1,4-galactan, and AG-II side chains. Radical scavenging assays and intracellular ROS production assay indicated. oligogalacturonides showed the best antioxidant activities compared with RG-II and RG-I domains, and the antioxidant activity of WLJP-A0.2b was due to the synergistic effects of different pectin domains. Therefore, pectic polysaccharides especially oligogalacturonides from *Lonicera japonica* Thunb. could be potential candidates as natural antioxidants and applied in functional food.

## Data availability statement

The original contributions presented in this study are included in the article/Supplementary material, further inquiries can be directed to the corresponding authors.

## Author contributions

XQ and YY: investigation and writing – original draft. XYW, JX, and XW: investigation. ZF: formal analysis. YZ: modifying the manuscript. HX and LS: writing – review and editing. All authors contributed to the article and approved the submitted version.
