# Supplementary material for: Structural characterization and anti-oxidation activity evaluation of pectin from Lonicera japonica Thunb
Source: Front Nutr. 2022 Sep 20;9:998462. doi: 10.3389/fnut.2022.998462 (PMC9530389; doi:10.3389/fnut.2022.998462)
Supplement: Supplementary file 1 [file Data_Sheet_1.docx]

Supplementary Material

**1 Supplementary Figures**

**Supplementary Figure S1**


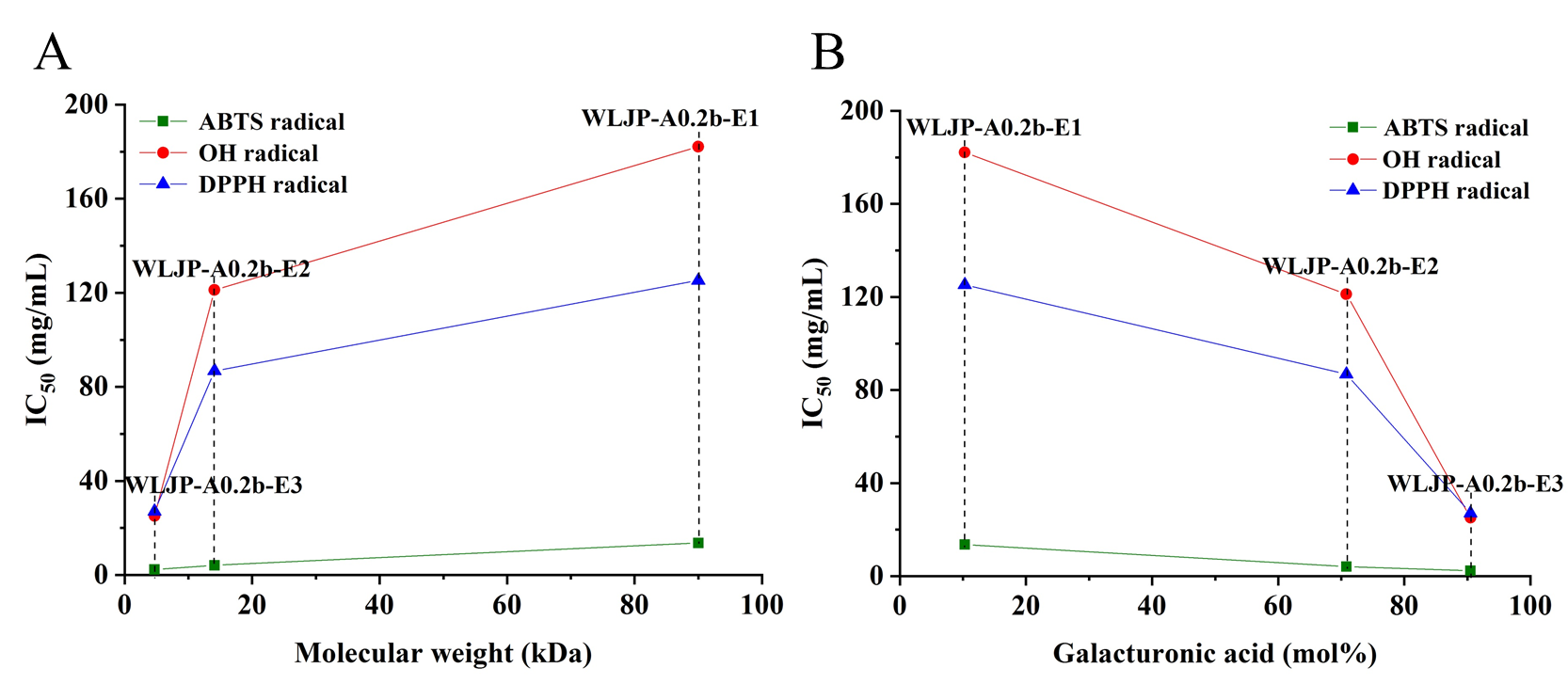


**Figure S1** (A) Correlation analysis between molecular weight and antioxidant activities of hydrolysis products of WLJP-A0.2b, (B) Correlation analysis between the content of galacturonic acid and antioxidant activities of hydrolysis products of WLJP-A0.2b.

**Supplementary Figure S2**

**
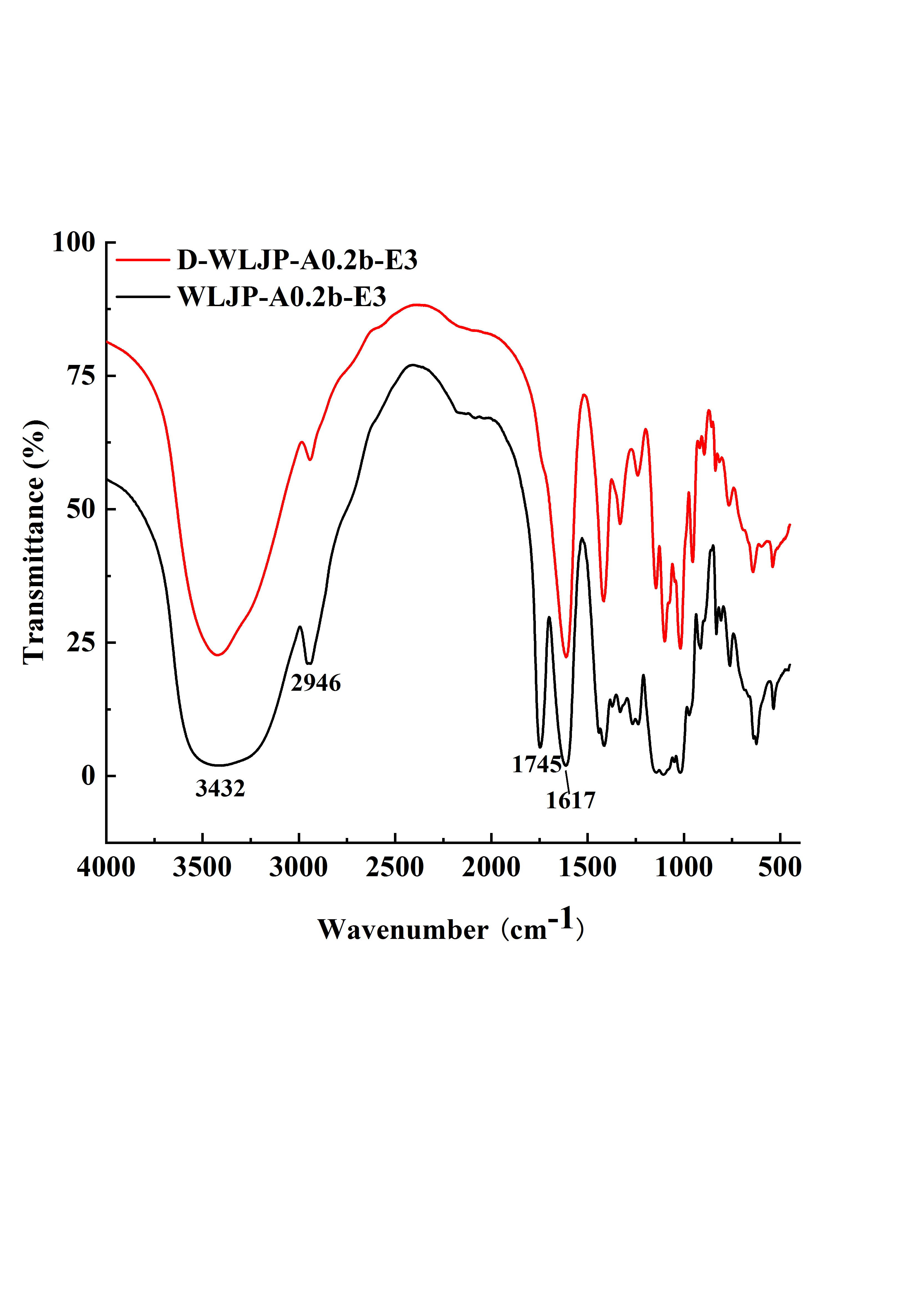
**

**Figure S2** FT-IR spectra of WLJP-A0.2b-E3 and D-WLJP-A0.2b-E3. D: de-esterified pectin.

**Supplementary Figure S3**


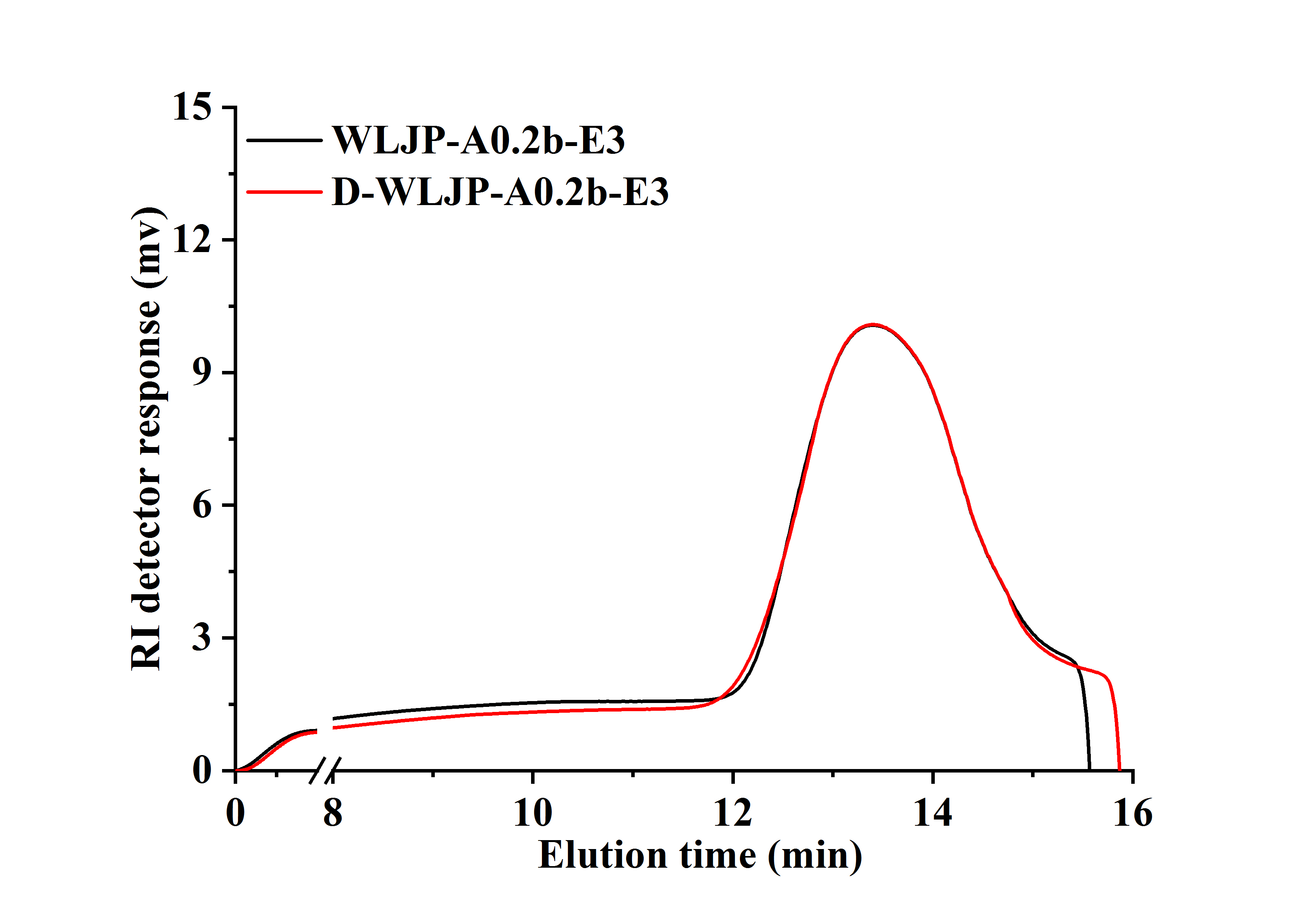


**Figure S3** The molecular weight distributions of WLJP-A0.2b-E3 and D-WLJP-A0.2b-E3.

**2 Supplementary Table**

**Supplementary Table S1** Chemical shifts assignments for C and H signals in NMR spectrum of WLJP-A0.2b

| **Residues** | **Chemical shift, δ(ppm)** | | | | | |
| --- | --- | --- | --- | --- | --- | --- |
|  | **C-1/H-1** | **C-2/H-2** | **C-3/H-3** | **C-4/H-4** | **C-5/H-5** | **C-6/H-6** |
| 1,4-α-Gal*p*A | 100.05/4.80 | 68.59/3.60 | 70.46/3.90 | 77.87/4.31 | 70.63/4.61 | 174.91 |
| →4)-α-GalpA(OMe)-(1→ | 100.68/4.86 | -- | 70.72/3.88 | 78.62/4.35 | 70.63/4.61 | 170.68 |
| 1,2-α-Rha*p* | 99.47/4.99 | 76.52/3.93 | 67.90/3.61 | -- | 68.59/3.60 | 16.22/1.16 |
| 1,2,4-α-Rha*p* | 98.99/4.99 | 76.52/3.93 | 67.90/3.61 | -- | 68.43/3.60 | 16.81/1.21 |
| Terminal β-Gal*p* | 103.35/4.37 | -- | -- | -- | -- | -- |
| 1,4-β-Gal*p* | 104.05/4.40 | 71.27/3.40 | 72.49/3.54 | 76.64/3.85 | 73.39/3.95 | 61.00/3.73 |
| 1,6-β-Gal*p* | 103.18/4.35 | 70.54/3.43 | 72.54/3.55 | 68.59/3.90 | -- | -- |
| 1,3-β-Gal*p* | 102.31/4.28 | -- | 79.73/4.19 | 68.44/3.88 | 73.44/3.95 | 61.10/3.61 |
| 1,3,6-β-Gal*p* | 103.35/4.37 | 71.27/3.40 | 80.22/4.20 | 68.59/3.90 | 73.44/3.95 | 69.93/3.90 |
| Terminal α-Ara*f* | 108.98/5.14 | 81.23/4.23 | 76.52/3.85 | 83.86/3.92 | 61.05/3.62 |  |
| 1,5-α-Ara*f* | 107.38/4.99 | 80.67/4.03 | 79.01/4.27 | 82.19/4.00 | -- |  |
| 1,3,5-α-Ara*f* | 107.06/5.04 | 78.29/4.28 | 83.86/3.98 | 81.23/4.20 | 66.43/3.84 |  |

**Supplementary Table S2** Chemical shifts assignments for C and H signals in NMR spectrum of WLJP-A0.2b-E1

| **Residues** | **Chemical shift, δ(ppm)** | | | | | |
| --- | --- | --- | --- | --- | --- | --- |
|  | **C-1/H-1** | **C-2/H-2** | **C-3/H-3** | **C-4/H-4** | **C-5/H-5** | **C-6/H-6** |
| 1,4-α-Gal*p*A | 98.60/4.92 | 67.54/3.65 | 70.68/3.96 | 78.31/4.30 | 71.22/4.80 | 173.64 |
| 1,2-α-Rha*p* | 97.47/5.00 | 76.06/3.97 | 68.59/3.98 | 70.49/3.38 | 67.73/3.79 | 16.46/1.08 |
| 1,2,4-α-Rha*p* | 97.47/5.00 | 76.06/3.97 | 68.59/3.98 | 74.79/3.53 | 66.44/3.73 | 16.78/1.15 |
| Terminal  β-Gal*p* | 102.64/4.33 | 71.23/3.68 | 72.92/3.63 | 76.05/3.97 | -- | 59.75/3.56 |
| 1,4-β-Gal*p* | 103.38/4.50 | 70.68/3.38 | 72.47/3.50 | 76.68/3.87 | 73.64/3.86 | 59.96/3.55 |
| 1,6-β-Gal*p* | 102.64/4.33 | -- | 71.64/3.98 | 67.95/3.98 | 73.30/3.77 | 69.23/4.11 |
| 1,3-β-Gal*p* | 102.60/4.32 | -- | 80.17/3.57 | 70.68/3.96 | 73.35/3.76 | 61.18/3.67 |
| 1,3,6-β-Gal*p* | 103.10/4.49 | 71.24/3.92 | 80.17/3.57 | 67.75/3.99 | 73.35/3.76 | 69.23/4.11 |
| Terminal  α-Ara*f* | 109.26/5.08 | 80.83/3.97 | 76.67/3.87 | 83.86/3.87 | 61.04/3.67 |  |
| 1,5-α-Ara*f* | 107.38/4.93 | 80.75/3.98 | 79.02/4.13 | 82.26/3.94 | 66.80/3.73 |  |
| 1,3,5-α-Ara*f* | 107.06/4.94 | 80.85/3.97 | 83.81/3.88 | 81.23/4.15 | 66.44/3.79 |  |

**Supplementary Table S3** m/z values and proposed structures of WLJP-A0.2b-E3 oligomers determined by ESI-MS

| ***m/z*** | **Proposed structure** | |
| --- | --- | --- |
| 193^1-^ | 1^00^ | GalA |
| 411^1-^ | 2^01^ | GalA GalA +Ac |
| 369^1-^ | 2^00^ | GalA GalA |
| 559^1-^ | 3^10^ | GalA GalA GalA+Me |
| 587^1-^ | 3^01^ | GalA GalA GalA +Ac |
| 749^1-^ | 4^20^ | GalA GalA GalA GalA+2Me |
| 469^2-^ | 5^30^ | GalA GalA GalA GalA GalA +3Me |
| 777^1-^ | 4^11^ | GalA GalA GalA GalA+Me+Ac |
| 545^1-^ | 3^00^ | GalA GalA GalA |
| 367^2-^ | 4^10^ | GalA GalA GalA GalA+Me |
| 462^2-^ | 5^20^ | GalA GalA GalA GalA GalA+2Me |
| 557^2-^ | 6^30^ | GalA GalA GalA GalA GalA GalA+3Me |
| 652^2-^ | 7^40^ | GalA GalA GalA GalA GalA GalA GalA+4Me |
| 747^2-^ | 8^50^ | GalA GalA GalA GalA GalA GalA GalA GalA+5Me |
| 571^2-^ | 6^21^ | GalA GalA GalA GalA GalA GalA+2Me+Ac |
| 455^2-^ | 5^10^ | GalA GalA GalA GalA GalA+Me |
| 550^2-^ | 6^20^ | GalA GalA GalA GalA GalA GalA+2Me |
| 645^2-^ | 7^30^ | GalA GalA GalA GalA GalA GalA GalA+3Me |
| 740^2-^ | 8^40^ | GalA GalA GalA GalA GalA GalA GalA GalA+4Me |
